# Angiotensin-(1–7) in Paraventricular Nucleus Modulates Sympathetic Activity and Cardiac Sympathetic Afferent Reflex in Renovascular Hypertensive Rats

**DOI:** 10.1371/journal.pone.0048966

**Published:** 2012-11-05

**Authors:** Ying Han, Hai-Jian Sun, Peng Li, Qing Gao, Ye-bo Zhou, Feng Zhang, Xing-Ya Gao, Guo-Qing Zhu

**Affiliations:** Key Laboratory of Cardiovascular Disease and Molecular Intervention, Department of Physiology, Nanjing Medical University, Nanjing, Jiangsu, China; Universidade Federal do Rio de Janeiro, Brazil

## Abstract

**Background:**

Excessive sympathetic activity contributes to the pathogenesis and progression of hypertension. Enhanced cardiac sympathetic afferent reflex (CSAR) is involved in sympathetic activation. This study was designed to determine the roles of angiotensin (Ang)-(1–7) in paraventricular nucleus (PVN) in modulating sympathetic activity and CSAR and its signal pathway in renovascular hypertension.

**Methodology/Principal Findings:**

Renovascular hypertension was induced with two-kidney, one-clip method. Renal sympathetic nerve activity (RSNA) and mean arterial pressure (MAP) were recorded in sinoaortic-denervated and cervical-vagotomized rats with anesthesia. CSAR was evaluated with the RSNA and MAP responses to epicardial application of capsaicin. PVN microinjection of Ang-(1–7) and cAMP analogue db-cAMP caused greater increases in RSNA and MAP, and enhancement in CSAR in hypertensive rats than in sham-operated rats, while Mas receptor antagonist A-779 produced opposite effects. There was no significant difference in the angiotensin-converting enzyme 2 (ACE2) activity and Ang-(1–7) level in the PVN between sham-operated rats and hypertensive rats, but the Mas receptor protein expression in the PVN was increased in hypertensive rats. The effects of Ang-(1–7) were abolished by A-779, adenylyl cyclase inhibitor SQ22536 or protein kinase A (PKA) inhibitor Rp-cAMP. SQ22536 or Rp-cAMP reduced RSNA and MAP in hypertensive rats, and attenuated the CSAR in both sham-operated and hypertensive rats.

**Conclusions:**

Ang-(1–7) in the PVN increases RSNA and MAP and enhances the CSAR, which is mediated by Mas receptors. Endogenous Ang-(1–7) and Mas receptors contribute to the enhanced sympathetic outflow and CSAR in renovascular hypertension. A cAMP-PKA pathway is involved in the effects of Ang-(1–7) in the PVN.

## Introduction

Sympathetic activity is enhanced in patients with essential [1] or secondary hypertension [2]–[5] and various hypertensive models [6]–[10]. Excessive sympathetic activity contributes to the pathogenesis of hypertension and progression of organ damage [11]–[13]. Intervention of the sympathetic activation is considered to be an antihypertensive strategy [14]–[16]. Cardiac sympathetic afferent reflex (CSAR) is known as a positive-feedback, sympathoexcitatory cardiovascular reflex [17], [18]. Previous studies in our lab have shown that the CSAR is enhanced in renovascular hypertensive rats [6], [19] and spontaneously hypertensive rats (SHR) [10], which contributes to the sympathetic activation and hypertension [20], [21].

Paraventricular nucleus (PVN) is an important component of the central neurocircuitry of the CSAR [22] and plays a major role in the integration of sympathetic outflow and cardiovascular activity via projections to the intermediolateral column (IML) of the spinal cord and the rostral ventrolateral medulla (RVLM) [23]. Angiotensin (Ang)-(1–7) is known as an important biological active peptide of renin–angiotensin system (RAS) family. Angiotensin-converting enzyme 2 (ACE2) hydrolyzes Ang II or Ang I to Ang-(1–7). Many of Ang-(1–7) effects are primarily mediated by Mas receptors [24] and are selectively blocked by its specific antagonist D-Alanine-Ang-(1–7) (A-779) [25]. Ang-(1–7) immunoreactive staining is present in the PVN [26] including parvocellular and magnocellular subdivisions [27]. The endogenous Ang-(1–7) level in the hypothalamus of rats is comparable to Ang I and Ang II [28]. The Mas receptors are expressed predominantly in the mouse and rat brain and particularly in the forebrain [29]. It has been reported that blockade of endogenous Ang-(1–7) by microinjection of A-779 into the PVN reduces renal sympathetic tone in normal rats [30]. Ang-(1–7) level in hypothalamus is increased in aortic coarctation-induced hypertensive rats [31]. However, it is not known whether Ang-(1–7) in the PVN is involved in the excessive sympathetic activation and the enhanced CSAR in hypertension.

**Table 1:** Body weight, SBP, MAP and HR at the end of the 4th week.

	Sham	2K1C
n	94	94
Body Weight, g	328.5±1.8	323.4±1.7
SBP, mm Hg	118.7±1.2	169.3±1.6 *
MAP, mm Hg	91.5±1.0	138.4±1.6 *
HR, beats/min	356.1±4.7	361.8±4.2

Systolic blood pressure (SBP), mean arterial pressure (MAP) and heart rate (HR) were measured with a pressure transducer in the right carotid artery under anesthesia. Values are expressed as mean ± SE. * P<0.05 compared with the Sham rats.

It has been found that activation of Mas receptors by Ang-(1–7) increases intracellular cAMP level and activates protein kinase A (PKA), while inhibition of either adenylyl cyclase (AC) or PKA activity attenuates Ang-(1–7)-induced ERK1/2 activation in glomerular mesangial cells [32]. Ang-(1–7) inhibits vascular growth through the prostacyclin-mediated production of cAMP and activation of cAMP-dependent PKA [33]. These results show that the cAMP-PKA signaling pathway is involved in the activity of Ang-(1–7) and Mas receptors in some peripheral tissues. However, whether cAMP-PKA pathway in the PVN is involved in the CSAR and sympathetic activation and the effects of Ang-(1–7) in hypertension is not understood. The present study was designed to determine whether Ang-(1–7) in the PVN contributed to the enhanced CSAR and sympathetic activation, and whether the cAMP-PKA pathway in the PVN was involved in the effects of Ang-(1–7) in hypertension.

**Figure 1:**
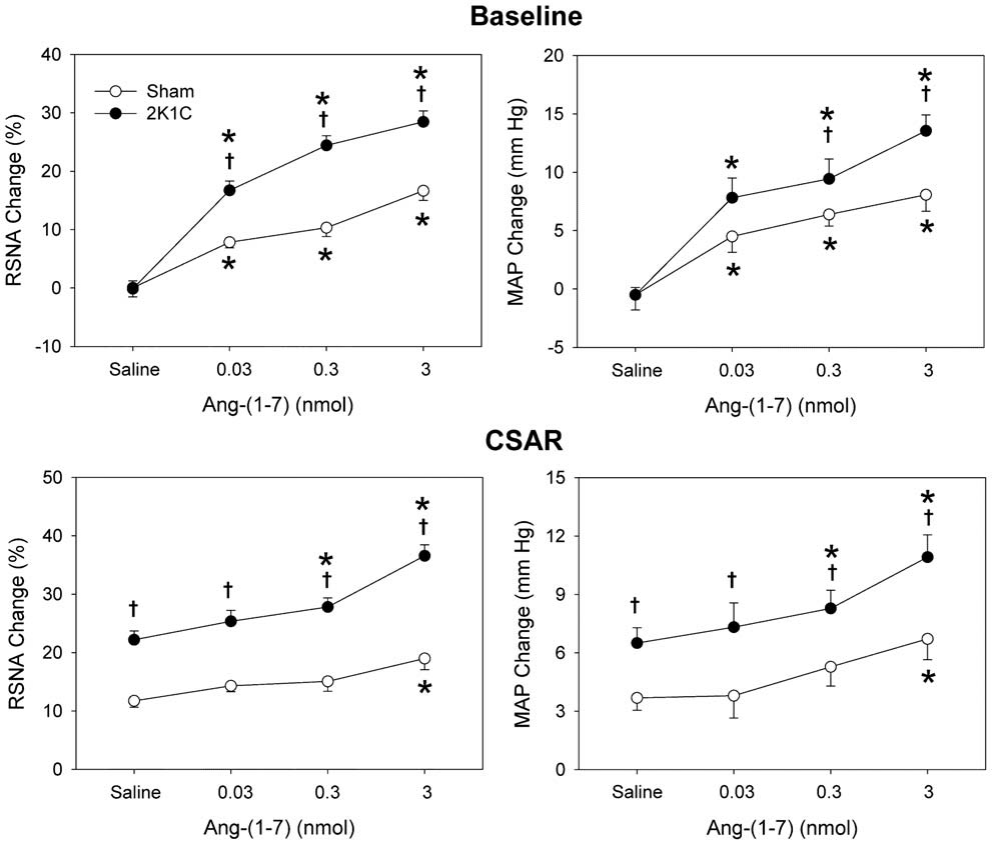
Effects of PVN microinjection of saline and three doses of Ang-(1–7) (0.03, 0.3 and 3 nmol) on the baseline RSNA and MAP and CSAR. The CSAR was evaluated by the RSNA and MAP responses to epicardial application of capsaicin (1 nmol). Values are mean ± SE. * P<0.05 compared with saline. † P<0.05 compared with Sham. n = 6 for each group.

**Figure 2:**
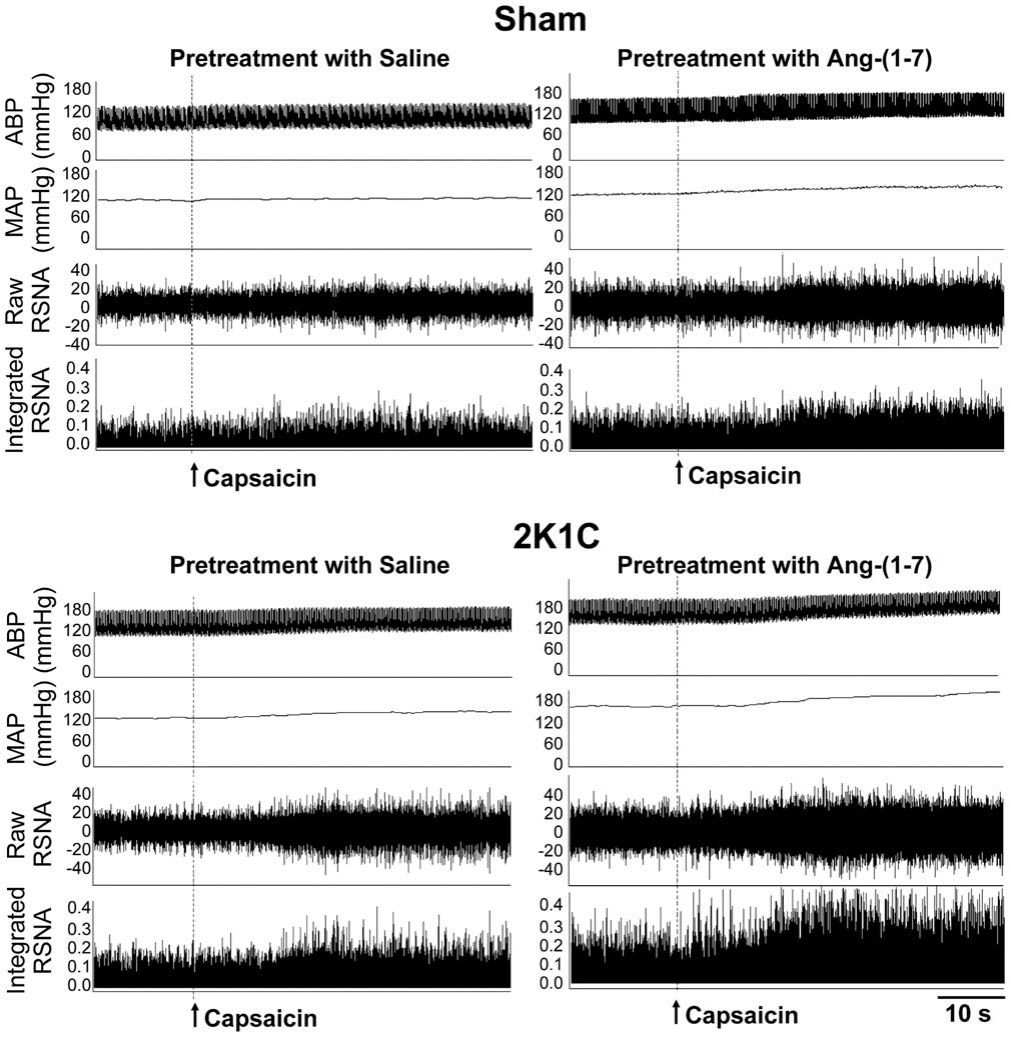
Representative recordings showing the effects of PVN microinjection of saline and Ang-(1–7) (3 nmol) on the CSAR in Sham and 2K1C rats. The CSAR was evaluated by the RSNA and MAP responses to epicardial application of capsaicin (1 nmol).

## Materials and Methods

Experiments were carried out in male Sprague–Dawley rats. The procedures were approved by the Experimental Animal Care and Use Committee of Nanjing Medical University (No. 20110316) and complied with the Guide for the Care and Use of Laboratory Animals (NIH publication no. 85–23, revised 1996). The rats were kept in a temperature-controlled room on a 12 h–12 h light–dark cycle with free access to standard chow and tap water.

**Figure 3:**
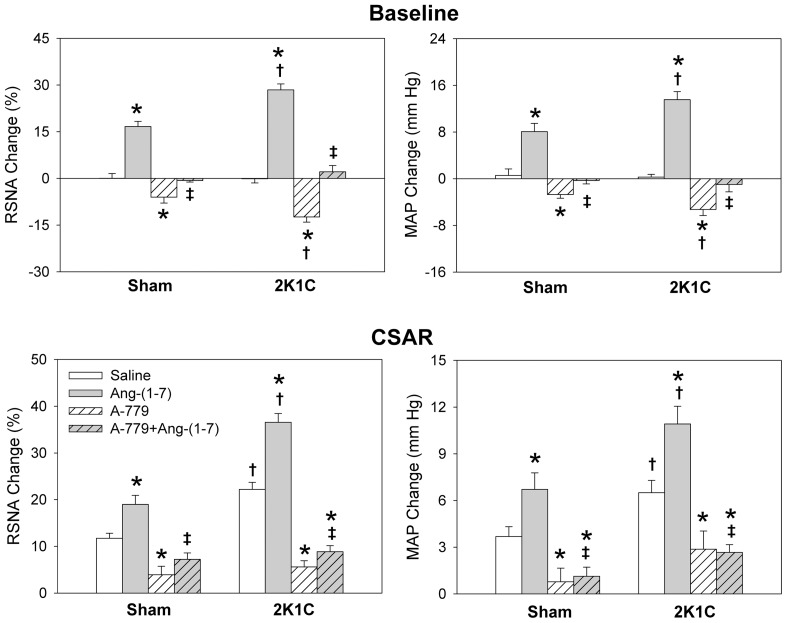
Effects of PVN microinjection of saline, Ang-(1–7) (3 nmol), A-779 (3 nmol), A-779+Ang-(1–7) on the baseline RSNA and MAP and CSAR. The CSAR was evaluated by the RSNA and MAP responses to epicardial application of capsaicin (1 nmol). Values are mean ± SE. * P<0.05 compared with saline. † P<0.05 compared with Sham. ‡ P<0.05 compared with Ang-(1–7) alone. n = 6 for each group.

### Renovascular hypertensive model

Goldblatt two-kidney one-clip (2K1C) method was used to induce renovascular hypertension in rats as previously reported [6], [20]. Simply, the rat weighing 160–180 g was anesthetized with intraperitoneal administration of sodium pentobarbital (50 mg kg^−1^). A retroperitoneal flank incision was performed to expose right renal artery. The artery was partly occluded by placing a U-shaped silver clip with an internal diameter of 0.20 mm on the artery to induce renovascular hypertension. Normotensive sham-operated (Sham) rat received similar surgical process except using silver clip. The criterion of hypertension is set as systolic blood pressure (SBP) of tail artery >160 mm Hg in conscious state [19], [20]. Six 2K1C rats were excluded, for their SBP was not high enough to meet the criterion.

**Figure 4:**
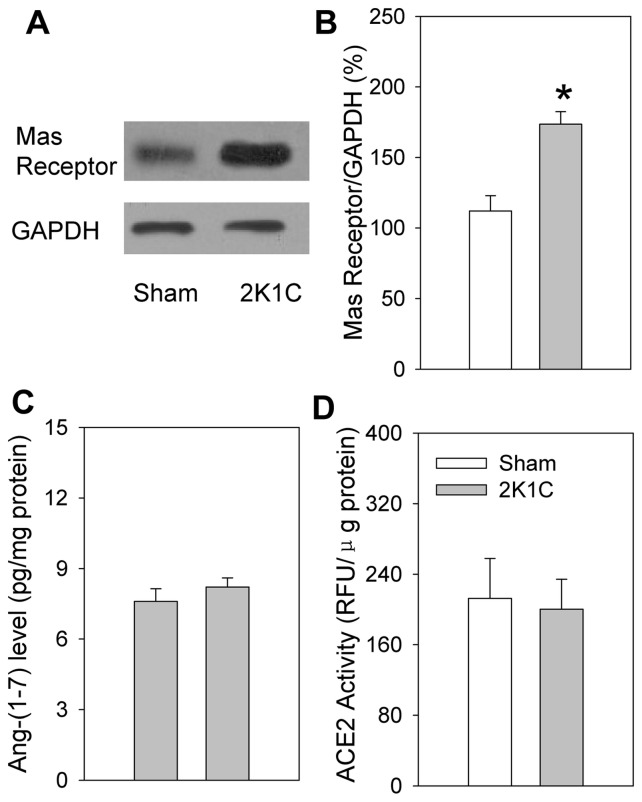
Mas receptor protein expression (A & B), Ang-(1–7) level (C) and ACE2 activity (D) in the PVN in Sham and 2K1C rats. RFU, relative fluorescence units. Values are mean ± SE. * P<0.05 compared with Sham rats. n = 5 for each group.

### SBP measurements in conscious state

Rats were trained by SBP measurement daily for at least 10 days before 2K1C or sham operation to minimize stress-induced SBP fluctuation. The SBP of tail artery was measured weekly in conscious state by using a noninvasive computerized tail-cuff system (NIBP, ADInstruments, Australia) [19], [20]. The rats were warmed for 10–20 min at 28°C before the measurements in order to allow the detection of tail artery pulsations and to achieve the pulse level ready. The SBP was obtained by averaging 10 measurements.

**Figure 5:**
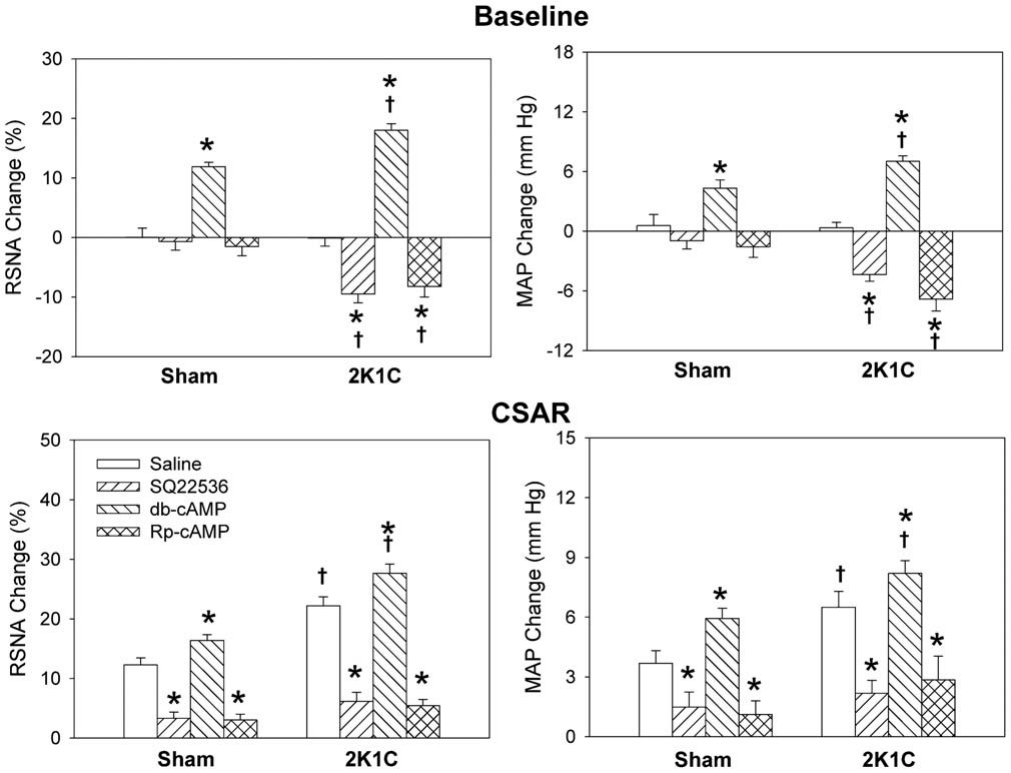
Effects of the PVN microinjection of saline, SQ22536 (2 nmol), db-cAMP (1 nmol) and Rp-cAMP (1 nmol) on the baseline RSNA and MAP and CSAR. The CSAR was evaluated by the RSNA and MAP responses to epicardial application of capsaicin (1 nmol). Values are mean ± SE. * P<0.05 compared with saline. † P<0.05 compared with Sham. n = 6 for each group.

**Figure 6:**
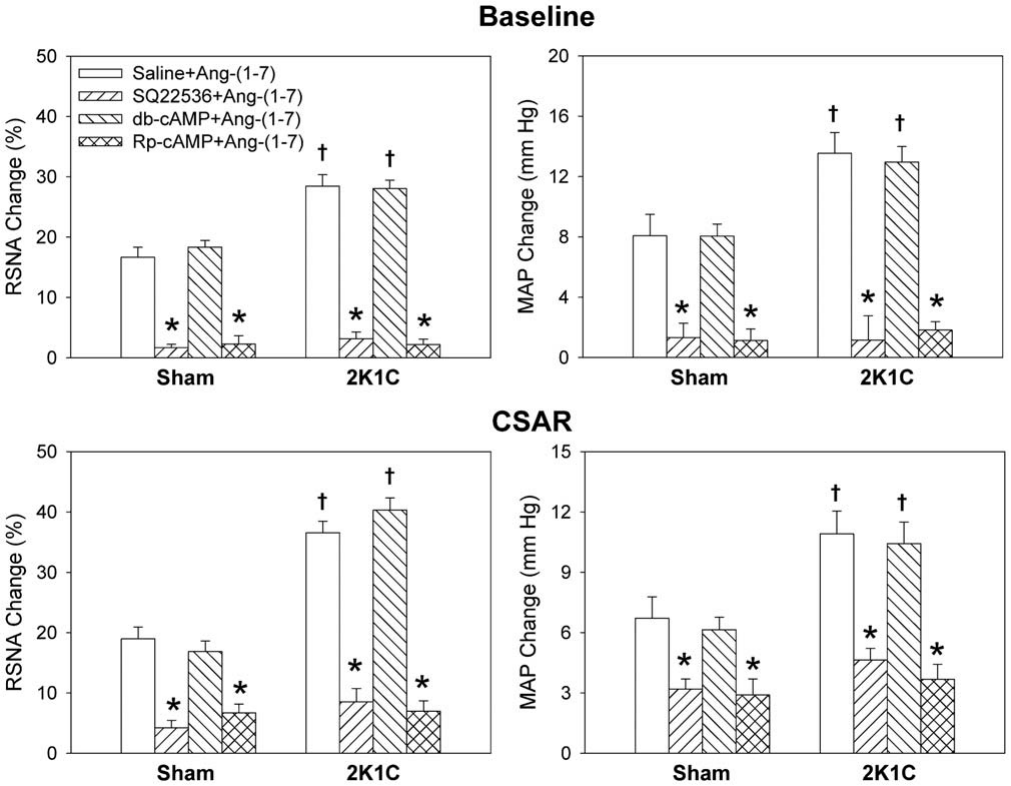
Effects of the PVN treatment with saline, SQ22536 (2 nmol), db-cAMP (1 nmol) and Rp-cAMP (1 nmol) on the RSNA, MAP and CSAR responses to Ang-(1–7) (3 nmol). The CSAR was evaluated by the RSNA and MAP responses to epicardial application of capsaicin (1 nmol). Values are mean ± SE. * P<0.05 compared with saline. † P<0.05 compared with Sham. n = 6 for each group.

### General procedures of acute experiment

Acute experiments were carried out at the end of the 4th week after the 2K1C or sham operation. Rat was intraperitoneally anesthetized with urethane (800 mg kg^−1^) and α-chloralose (40 mg kg^−1^). Supplemental doses of anesthesia were used to maintain an appropriate degree of anesthesia that was assessed by the absence of corneal reflex and paw withdrawal response to a noxious pinch. The rat was mechanically ventilated with room air using a rodent ventilator (model 683, Harved Apparatus Inc, USA). The right carotid artery was cannulated and connected with a pressure transducer (MLT0380, ADInstruments, Australia) for continuous recording of arterial blood pressure (ABP), mean arterial pressure (MAP) and heart rate (HR). Bilateral baroreceptor denervation and vagotomy were carried out and identified as previously reported [21], [34].

### Renal sympathetic nerve activity (RSNA) recordings

A retroperitoneal incision was made and the left renal sympathetic nerve was isolated. The renal nerve was cut distally to eliminate its afferent activity. The nerve was placed on a pair of silver electrodes and immersed in warm mineral oil. The nerve signals were amplified with a four channel AC/DC differential amplifier (DP-304, Warner Instruments, Hamden, CT, USA) with a high pass filter at 10 Hz and a low pass filter at 3,000 Hz. The RSNA was integrated at a time constant of 100 ms. At the end of each experiment, the background noise was determined after section of the central end of the nerve and was subtracted from the integrated values of the RSNA [35]. The raw and integrated RSNA, ABP, MAP and HR were simultaneously recorded with a PowerLab data acquisition system (8/35, ADInstruments, Australia).

### Evaluation of CSAR

A limited left lateral thoracotomy was performed to expose the heart and the pericardium was removed. The CSAR was induced by stimulating cardiac sympathetic afferents with epicardial application of a piece of filter paper (3 mm×3 mm) containing capsaicin (1.0 nmol in 2.0 μl) on the anterior wall of the left ventricle. The filter paper was removed 1 minute later, and the ventricular surface was rinsed three times with 10 ml of normal saline (37°C). The CSAR was evaluated by the RSNA and MAP responses to the epicardial application of capsaicin [34], [35].

### PVN microinjection

The rats were placed in a stereotaxic frame (Stoelting, Chicago, USA). The stereotaxic coordinates for the PVN are 1.8 mm caudal from bregma, 0.4 mm lateral to the midline and 7.9 mm ventral to the dorsal surface according to Paxinos & Watson's rat atlas. The bilateral PVN microinjections were completed within 1 min and the microinjection volume was 50 nL for each side of the PVN. At the end of the experiment, 50 nl of Evans Blue dye (2%) was injected into each microinjection site. The microinjection sites were histologically verified with microscope. Rats with microinjection sites outside the PVN were excluded from data analysis.

### PVN Sample Preparation

The rat was euthanized with an overdose of pentobarbital. The brain of the rat was quickly removed, frozen in liquid nitrogen and stored at −70°C until being sectioned. Coronal sections of the brain were made with a cryostat microtome (Leica CM1900-1-1, Wetzlar, Hessen, Germany) at the PVN level. The PVN area was punched out with a 15-gauge needle (1.5 mm ID). The punched tissues were subsequently homogenized and centrifuged. The total protein in the homogenate supernatant was extracted and measured by using protein assay kit (BCA; Pierce).

### Measurement of Mas receptor protein expression

The Mas receptor protein expression in the PVN was determined with Western blotting method [36]. Briefly, after process of electrophoresis and transmembrane, proteins on nitrocellulose membrane were probed with rabbit polyclonal Mas receptor antibody (1∶200, Alomone Labs, Israel). This was followed by incubation with horseradish peroxidase–conjugated goat anti-rabbit IgG (1∶5000; Immunology Consultants Lab, USA). The bands were visualized by enhanced chemiluminescence using the ECL system (Pierce Chemical). GAPDH (Bioworld Technology Inc., USA) protein was used as a loading control. The total amount of Mas receptors protein is expressed as the percentage of Mas receptors to GAPDH protein.

### Measurement of Ang-(1–7) level

The level of Ang-(1–7) in PVN tissue homogenate supernatant was measured using a commercial peptide enzyme immunoassay kit (MyBioSource LLC, USA) following the manufacturer's instructions [37].

### Measurement of ACE2 activity

ACE2 activity in the PVN was determined by a fluorimetric ACE2 activity assay kit (Anaspec Inc. Fremont, CA, USA). The assay is based on the use of the ACE2 substrate Mca/Dnp fluorescence resonance energy transfer (FRET) peptide. In the FRET peptide the fluorescence of Mca is quenched by Dnp. Upon cleavage into two separate fragments by the ACE2 enzyme, the fluorescence of Mca is recovered, and can be monitored at excitation/emission = 330 nm/390 nm. Briefly, PVN tissue homogenate supernatant was added to a black flat-bottom 96-well plate containing ACE2 substrate [37], [38]. The change in fluorescence was monitored using a Fluorescence Reader (Gemini EM, Molecular Devices, USA). All fluorescence readings are expressed in relative fluorescence units (RFU) and the ACE2 activity is expressed as amounts of RFU of substrate converted to product per unit time and is normalized for protein content [39], [40].

### Chemicals

Ang-(1–7) and A-779 were purchased from Bachem (Bubendorf, Switzerland). Dibutyryl-cAMP (db-cAMP), 9-(tetrahydro-2-furanyl)-9H-purin-6-amine (SQ22536), rp-adenosine-3′,5′-cyclic monophosphothionate (Rp-cAMP) and capsaicin were purchased from Sigma Chemical Co (St. Louis, MO, USA). All the chemicals were dissolved in normal saline.

### Experiment protocols

#### Experiment 1

The PVN microinjection of saline, three doses of Ang-(1–7) (0.03, 0.3 and 3 nmol), Mas receptor antagonist A-779 (3 nmol) and Ang-(1–7) (3 nmol) pretreated with A-779 (3 nmol) on the RSNA, MAP and CSAR were carried out in six groups of 2K1C rats and six groups of Sham rats, respectively (n = 6 for each group). A-779 was administered eight min before Ang-(1–7). The dose and duration of treatment with A-799 were selected according to our preliminary study and previous reports [30], [41]. The CSAR was evaluated eight min after the PVN microinjection. To exclude the possibility that the effects of Ang-(1–7) were caused by diffusion to other brain area, the effects of microinjection of Ang-(1–7) (3 nmol) into the anterior hypothalamic area which is adjacent to the PVN were determined in 2K1C and Sham rats (n = 3 for each group).

#### Experiment 2

Mas receptor protein expression in the PVN was determined in 5 Sham rats and 5 2K1C rats. Furthermore, Ang-(1–7) level and ACE2 activity in the PVN were determined in other 5 Sham rats and 5 2K1C rats.

#### Experiment 3

PVN microinjection of saline, SQ22536 (2 nmol, an AC inhibitor), db-cAMP (1 nmol, a cAMP analogue) or Rp-cAMP (1 nmol, a PKA inhibitor) were carried out in four groups of Sham rats and four groups of 2K1C rats to determine the roles of cAMP and PKA in regulating RSNA, MAP and CSAR (n = 6 for each group). In other four groups of Sham rats and four groups of 2K1C rats, PVN pretreatment with the same dose of saline, SQ22536, db-cAMP or Rp-cAMP on the RSNA, MAP and CSAR responses to Ang-(1–7) (3 nmol) were determined (n = 6 for each group). The pretreatment was done eight min before Ang-(1–7). CSAR was evaluated eight min after the PVN microinjection of Ang-(1–7).

### Statistical analysis

The RSNA and MAP responses caused by the PVN microinjection were determined by averaging 2 min of the maximal responses. The RSNA and MAP responses to epicardial application of capsaicin were determined by averaging 30 sec of the parameters beginning at the 16th sec after epicardial application of capsaicin. The RSNA change was expressed as the percent change from the baseline values. Comparisons between two observations in the same animal were assessed by Student's paired *t* test. One-way or two-way ANOVA was used followed by Bonferroni test for post hoc analysis when multiple comparisons were made. All data were expressed as mean ± SE. *P*<0.05 was considered statistically significant.

## Results

### General Data

SBP and MAP in 2K1C rats were significantly higher than that in Sham rats. There was no significant difference in the body weight and baseline HR between Sham rats and 2K1C rats (Table 1).

### Effects of different doses of Ang-(1–7)

Microinjection of three doses of Ang-(1–7) (0.03, 0.3 and 3 nmol) into the PVN dose-related increased the baseline RSNA and MAP in both 2K1C rats and Sham rats, peaking at about 7 min and lasting at least 12 min. The effects of Ang-(1–7) on RSNA and MAP in 2K1C rats were greater than that in Sham rats (Fig. 1). Representative recordings showed that the CSAR was enhanced in 2K1C rat compared with Sham rat, and PVN microinjection of high dose of Ang-(1–7) enhanced the CSAR compared with saline in both Sham rat and 2K1C rat (Fig. 2). Compared with saline, high dose of Ang-(1–7) significantly enhanced the CSAR in Sham rats, while both moderate and high doses of Ang-(1–7) enhanced the CSAR in 2K1C rats (Fig. 1). In addition, microinjection of high dose of Ang-(1–7) into the anterior hypothalamic area had no significant effects on the RSNA, MAP and CSAR.

### Effects of A-779

Microinjection of A-779 into the PVN decreased the RSNA and MAP, peaking at about 8 min and lasting at least 30 min, and attenuated the CSAR in both Sham and 2K1C rats. The effects of A-779 were greater in 2K1C rats than that in Sham rats. Pretreatment with A-779 in the PVN abolished the effects of Ang-(1–7) on the RSNA, MAP and CSAR in Sham and 2K1C rats (Fig. 3).

### Mas receptor protein expression, Ang-(1–7) level and ACE2 activity in PVN

The Mas receptor protein expression in the PVN was significantly increased in 2K1C rats compared with Sham rats. However, there were no significant difference in Ang-(1–7) level and ACE2 activity in the PVN between 2K1C rats and Sham rats (Fig. 4).

### Effects of SQ22536 and db-cAMP

Microinjection of AC inhibitor SQ22536 into the PVN decreased RSNA and MAP in 2K1C rats, and attenuated the CSAR in both Sham and 2K1C rats. A cAMP analogue db-cAMP caused greater increases in RSNA and MAP, and enhancement in CSAR in 2K1C rats than in Sham rats (Fig. 5). On the other hand, pretreatment with SQ22536 in the PVN abolished the effects of Ang-(1–7) on the RSNA, MAP and CSAR in both Sham and 2K1C rats, but db-cAMP pretreatment failed to augment the effects of Ang-(1–7) (Fig. 6).

### Effects of Rp-cAMP

Microinjection of PKA inhibitor Rp-cAMP into the PVN decreased RSNA and MAP in 2K1C rats, and attenuated the CSAR in both Sham and 2K1C rats (Fig. 5). Pretreatment with Rp-cAMP in the PVN abolished the effects of Ang-(1–7) on the RSNA, MAP and CSAR in both Sham and 2K1C rats (Fig. 6).

## Discussion

The primary new findings in the present study are that activation of Mas receptors with Ang-(1–7) in the PVN increases sympathetic outflow and blood pressure, and augments the CSAR. Endogenous Ang-(1–7) and Mas receptors contribute to the excessive sympathetic activation and the enhanced CSAR in renovascular hypertensive rats. A cAMP-PKA pathway is involved in the effects of Ang-(1–7) in the PVN.

Abundant Ang-(1–7) immunoreactive staining has been found in the PVN [26]. Microiontophoretic application of Ang-(1–7) into the PVN augments the excitability of the neurons in the PVN [42], which is selectively blocked by Mas receptor antagonist A-779 [43]. Blockade of endogenous Ang-(1–7) by microinjection of A-779 into the PVN reduces renal sympathetic tone in normal rats [30]. In the present study, microinjection of Ang-(1–7) into the PVN caused greater enhancement in the CSAR, and more increases in the RSNA and MAP in 2K1C rats than in Sham rats, which was abolished by A-779. A-779 in the PVN caused greater decreases in RSNA and MAP in 2K1C rats than in Sham rats, and attenuated the CSAR in both Sham and 2K1C rats. These results indicate that Ang-(1–7) in the PVN contributes to the enhanced sympathetic activity and CSAR via Mas receptors in renovascular hypertension. It is known that the CSAR is enhanced in 2K1C-induced hypertensive rats [6], [19] and SHR [10], which is involved in sympathetic activation and hypertension in these animal models [20], [21]. The inhibitory effects of A-779 on the CSAR may be partially responsible for the decreases in RSNA and MAP in renovascular hypertension.

Many studies have shown that Ang-(1–7)/Mas receptor axis is as a counter regulator of the effects of the classic Ang II/AT_1_ receptor axis-mediated effects [44]–[46]. However, a previous study in our lab has indicated that Ang-(1–7) in the RVLM is as effective as Ang II in sensitizing the CSAR and increasing sympathetic outflow in normal rats [41]. The results is confirmed by a recent finding that endogenous Ang-(1–7) in the RVLM contributes to maintain arterial blood pressure and renal sympathetic nerve activity in both SHR and normal control rats [47]. We have found that Ang II in the PVN augmented the enhanced CSAR and RSNA in 2K1C rats, which were abolished by the pretreatment with AT_1_ receptor antagonist losartan, and losartan in the PVN normalized the enhanced CSAR and decreased the RSNA and MAP in 2K1C rats [21]. The results in the present study indicate that the roles of Ang-(1–7) are similar to Ang II in modulating the RSNA MAP and CSAR. However, there are some differences between Ang II/AT_1_ receptors and Ang-(1–7)/Mas receptors in the PVN in regulating RSNA, MAP and CSAR. In normal rats, AT_1_ receptor antagonist losartan in the PVN had no significant effects on RSNA and MAP [21], but Mas receptors antagonist A-779 decreased RSNA and MAP. The results suggest that Mas receptors rather than AT_1_ receptors in the PVN are involved in the tonic control of sympathetic activity and blood pressure. Furthermore, losartan had no significant effects on the CSAR in normal rats and normalized the enhanced CSAR in 2K1C rats to the baseline level [21], but A-779 reduced the CSAR in both Sham and 2K1C rats to the lower level than baseline. These results suggest that Ang-(1–7)/Mas receptors rather than Ang II/AT_1_ receptors in the PVN contribute to the tonic control of the CSAR. Blockade of Mas receptors in the PVN caused greater CSAR-inhibitory effect in 2K1C rats.

We found that the Mas receptor protein expression in the PVN was significantly increased in 2K1C rats compared with Sham rats. However, there were no significant difference in Ang-(1–7) level and ACE2 activity in the PVN between 2K1C rats and Sham rats. These results suggest that the enhanced effects of Ang-(1–7) in the PVN on the RSNA, MAP and CSAR in 2K1C rats arise from the upregulation of Mas receptors in the PVN rather than the production or release of Ang-(1–7) in the PVN. The increased Mas receptor expression in the PVN contributes to the tonic control of RSNA, MAP and CSAR in 2K1C rats.

It is known that nitric oxide (NO) is a key signaling molecule in Ang-(1–7) stimulated neurons that may counteract the signaling events of Ang II [48]. NO in the PVN inhibits the Ang II-mediated increase in sympathetic nerve activity [49]–[52]. The findings in the present study showed that the roles of Ang-(1–7) in the PVN in increasing the RSNA and MAP, and enhancing the CSAR were similar to the roles of Ang II as previously reported [21]. Therefore, it would be impossible that nitric oxide mediate the excitatory effects of Ang-(1–7) in the PVN on the RSNA, MAP and CSAR. The Mas receptor is a kind of G protein-coupled receptor [53]. Ang-(1–7) inhibits vascular growth through the prostacyclin-mediated production of cAMP and activation of cAMP-dependent PKA [33]. Ang-(1–7)-induced activation of ERK1/2 in glomerular mesangial cells is cAMP/PKA-dependent [32]. It is an interesting question whether cAMP/PKA in the PVN is involved in mediating the effects of Ang-(1–7) in the PVN.

In the present study, microinjection of AC inhibitor SQ22536 or PKA inhibitor Rp-cAMP into the PVN decreased RSNA and MAP in 2K1C rats, and attenuated the CSAR in both Sham and 2K1C rats. Pretreatment with SQ22536 or Rp-cAMP in the PVN abolished the effects of Ang-(1–7) on the RSNA, MAP and CSAR in both Sham and 2K1C rats. These results indicate that the cAMP-PKA pathway in the PVN mediates the effects of Ang-(1–7) on the RSNA, MAP and CSAR, and is involved in the sympathetic activation and enhanced CSAR in renovascular hypertension. SQ22536 or Rp-cAMP reduced the CSAR in both Sham and 2K1C rats to the lower level than baseline, suggesting that cAMP in the PVN contributes to the tonic control of the CSAR. The results is supported by the findings that PVN microinjection of a cAMP analogue db-cAMP caused greater increases in RSNA and MAP, and enhancement in CSAR in 2K1C rats than in Sham rats.

In conclusion, activation of Mas receptors with Ang-(1–7) in the PVN increases sympathetic outflow and blood pressure, and augments the CSAR. Endogenous Ang-(1–7) and Mas receptors contribute to the excessive sympathetic activation and the enhanced CSAR in renovascular hypertensive rats. A cAMP-PKA pathway is involved in the excitatory effects of Ang-(1–7) on the sympathetic activity, blood pressure and CSAR in the PVN. Blockade of Mas receptors or the cAMP-PKA pathway in the PVN may be beneficial to attenuate the excessive sympathetic activation in hypertension.
